# Risk of Hospitalization for Community Acquired Pneumonia with Renin-Angiotensin Blockade in Elderly Patients: A Population-Based Study

**DOI:** 10.1371/journal.pone.0110165

**Published:** 2014-10-29

**Authors:** Sachin Shah, Eric McArthur, Alexandra Farag, Michael Nartey, Jamie L. Fleet, Greg A. Knoll, S. Joseph Kim, Amit X. Garg, Arsh K. Jain

**Affiliations:** 1 Division of Nephrology, Department of Medicine, Western University, London, Ontario, Canada; 2 Department of Epidemiology and Biostatistics, Western University, London, Ontario, Canada; 3 Institute for Clinical Evaluative Sciences, London, Ontario, Canada; 4 Division of Nephrology, Kidney Research Centre, Ottawa Hospital Research Institute, University of Ottawa, Ottawa, Ontario, Canada; 5 Division of Nephrology, Toronto General Hospital, University Health Network, University of Toronto, Toronto, Ontario, Canada; Osaka University Graduate School of Medicine, Japan

## Abstract

**Objective:**

To characterize the 90-day risk of hospitalization with pneumonia among patients treated with different anti-hypertensive drug classes.

**Design:**

Population based cohort study using five linked databases.

**Participants:**

Individuals over the age of 65 who filled a new outpatient prescription for one of four anti-hypertensive medications: ACE inhibitors (n = 86 775), ARBs (n = 33 953), calcium channel blockers (CCB, n = 34 240), beta blockers (BB, n = 35 331) and thiazide diuretics (n = 64 186).

**Primary Outcome:**

Hospitalization with pneumonia within 90 days of a qualifying prescription. We adjusted for ten *a priori* selected covariates, including age, sex, diabetes and number of visits to a family doctor.

**Results:**

Baseline characteristics of the groups were relatively well matched, except for age, sex, diabetes and frequency of family doctor visits. 128 of the 86 775 patients (0.15%) initiated on an ACE inhibitor and 43 of the 33953 patients (0.13%) of patients initiated on an ARB were hospitalized with pneumonia in the subsequent 90 days. 135 of 64 186 patients (0.21%) initiated on a thiazide, 112 of 35 331 patients (.32%) initiated on a BB, and 89 of 34 240 (0.26%) patients initiated on a CCB achieved the primary outcome. Compared to calcium channel blockers, ACE inhibitors (adjusted OR 0.61, 95% CI 0.46 to 0.81) and ARBs (adjusted OR 0.52, 95% CI 0.36 to 0.76) were associated with a lower risk of pneumonia. No benefit was seen with thiazides (adjusted OR 0.87, 95% CI 0.66 to 1.14) or beta blockers (adjusted OR 1.21, 95% CI 0.91 to 1.60).

**Conclusion:**

Initiating medications that block the renin angiotensin system, compared to other anti-hypertensive medications, is associated with a small absolute reduction in the 90 day risk of hospitalization with pneumonia.

## Introduction

Community-acquired pneumonia (CAP) is commonly encountered in clinical practice and its incidence increases with age [1], [2]. It is the eighth leading cause of death in Canada and the United States and the leading cause of infection-related hospitalization [3]. Following hospitalization for pneumonia, 30-day mortality rates have been reported as high as 23% [3]. This significant clinical burden has prompted attempts to identify strategies that may reduce the incidence of CAP. Specifically, there is a growing body of literature demonstrating a reduced incidence of pneumonia in patients treated with angiotensin converting enzyme (ACE) inhibitors [4]–[8]. While not fully understood, the proposed mechanism by which ACE inhibitors may protect against pneumonia is related to improvement in both cough and swallowing reflexes, an effect thought to be mediated through increased levels of substance P and bradykinins [9]–[14].

Recently, a meta-analysis of randomized and non-randomized studies demonstrated a reduced risk of pneumonia in ACE inhibitor users [6]. No benefit was seen with ARBs, which is consistent with the proposed mechanism, as this class of medication does not affect substance P and bradykinin levels [15]. However, rather interestingly, there was a trend towards a reduced risk of pneumonia with ARBs when only randomized trials were considered (odds ratio (OR) for pneumonia 0.9, 95% confidence interval (CI) 0.79 to 1.01).

Given these heterogeneous results, we conducted the current study to characterize the 90-day risk for hospitalization with pneumonia in a large population of older adults initiated on ACE inhibitors, ARBs, beta blockers (BB) or thiazides in a routine outpatient care setting. We compared these patients to a similar group of older adults prescribed a calcium-channel blocker (CCB). We hypothesized that a reduction in the incidence of pneumonia would be seen with both ACE inhibitors and ARBs when compared to the CCBs, but no benefit would be seen with thiazides or BBs.

## Methods

### Ethics

We conducted this study according to a prespecified protocol that was approved by the Research Ethics Board at Sunnybrook Health Sciences Centre (Toronto, Ontario, Canada).

### Study Design and Setting

We conducted a population-based retrospective cohort study using health administrative data from Ontario, Canada. Ontario is Canada's most populous province with approximately 13 million residents who receive universal access to hospital and physician services (Statistics Canada). Ontario's 1.8 million residents over the age of 65 years also receive prescription drug coverage.

### Data Sources

We used five linked databases housed at the Institute for Clinical Evaluative Sciences to conduct this study. We ascertained vital statistics from the Registered Persons Database (RPDB). The RPDB records the demographic information for people issued a provincial health card. We used the Ontario Drug Benefits (ODB) database to ascertain prescription drug exposure, including the independent variables and drug-related baseline characteristics. The ODB records prescription drug use for patients over the age of 65 years (a universal benefit) and has an error rate of <1% [16]. We identified admissions to hospital and baseline characteristics using the Canadian Institute for Health Information Discharge Abstract Database (CIHI-DAD). The CIHI-DAD records hospital admissions and related diagnostic and procedural information. For similar information relating to emergency department admissions, we used the National Ambulatory Care Reporting System (NACRS) database. We used the Ontario Health Insurance Plan (OHIP) database to ascertain information relating to physician services.

### Patients

We restricted enrollment to patients over the age of 65 years who, between June 1, 2003 and December 31, 2011, filled a prescription for any of five drug classes: ACE inhibitor, ARB, CCB, BB or thiazide. We chose to use a new user design with 90 day outcomes in order to reduce the incidence of intervening health events that may have biased patient outcome. We excluded all individuals who, in the prior 180 days, were taking any type of anti-hypertensive, diuretic or anti-arrhythmic. In order to compare mutually exclusive groups, we excluded patients who had filled a prescription for more than one study drug class on the day the new prescription was filled. To help further limit confounding by indication, we excluded any conditions that may dictate use of one study drug over the other. These exclusions were baseline evidence of coronary artery disease or congestive heart failure or chronic kidney disease. All comorbidities were assessed in the five years preceding the qualifying prescription, except for chronic kidney disease (one year). Finally, to further reduce the risk of recent health events influencing prescription choice we excluded patients who were admitted to hospital in the 90 days preceding the qualifying prescription date, or visited an emergency department in the 30 days preceding the qualifying prescription date.

### Outcomes

All outcomes were assessed in the 90 days after initiation of one of the study drugs (ACE inhibitor, ARB, BB, thiazide or CCB). We chose 90 day outcomes for two reasons. First, the short duration of follow-up was felt to help mitigate against the risk of contamination (such as crossing-over from one study drug class to another or adding in a different study drug). Second, prescriptions covered by the provincial drug plan are prescribed at no more than 100-day intervals. We assessed the rates of contamination directly by looking at how many patients in each study group received an alternate study drug during follow-up.

#### Primary outcome: Admission to hospital with pneumonia

We defined the primary outcome of hospital admission with pneumonia as any admission with a J12-J18 or P23 International Classification of Diseases, 10^th^ revision (ICD-10) diagnostic code in any of the CIHI-DAD's diagnostic fields. In patients greater than 65 years of age who are in a hospitalized setting, similar codes have been shown to be extremely accurate in identifying cases of pneumonia (sensitivity and specificity about 98% and 97%, respectively).

In a sensitivity analysis, we restricted our primary outcome to patients whose primary reason for admission was pneumonia. We achieved this by using one of the aforementioned diagnostic codes, but restricting to the “admitdx” field only in the CIHI-DAD.

#### Sub-group analysis: Diabetes

Given the increased risk of pneumonia with diabetes and the possibility that these patients would not be equally distributed among the groups, we performed a sub-group analysis of patients with and without diabetes. We assessed for any effect modification of diabetes on the association of anti-hypertensive medication type and our primary outcome. Diabetics were identified as any patient who filled a prescription for a medication used to treat diabetes (insulin or oral hypoglycemic) in the 180 days preceding the qualifying prescription.

#### Tracer Outcome: Hospitalization with cholecystitis

We tested the specificity of our findings by determining each group's risk of cholecystitis, an outcome we did not expect to be influenced by the choice of anti-hypertensive medication. We defined cholecystitis as any admission 90 days following the prescription date with the ICD-10 diagnosis code K8000, K8001, K8010, K8011, K8040, K8041, K81 found in any of the CIHI-DAD's diagnostic fields. We also used provider claim diagnosis codes 574 and 575 submitted by healthcare providers.

### Statistical methods

We compared the prevalence of baseline characteristics between the medication groups (ACE, ARB, thiazides, BB and CCB) using standardized differences with CCB acting as the referent group. This metric describes differences between group means as a proportion of the pooled standard deviation. Larger values indicate larger differences and those greater than 10% are considered meaningful [17], [18].

The administrative datasets at the Institute for Clinical Evaluative Sciences allowed for essentially 100% follow-up on all patients during the 90-day risk period. We used a multivariable logistic regression model to adjust for the following ten covariates selected *a priori*: age, sex, number of different visits to a family doctor in the one year preceding the prescription date, Charlson Co-morbidity Index Score, chronic liver disease, pneumonia, chronic lung disease (including asthma and COPD), stroke, diabetes mellitus, and dementia. Baseline characteristics were assessed in the five years preceding the qualifying prescription. We conducted all analysis with SAS 9.2 (SAS Institute Incorporated, Cary, North Carolina, USA, 2008). A two-sided *P* value of <0.05 was considered statistically significant.

## Results

**Figure 1:**
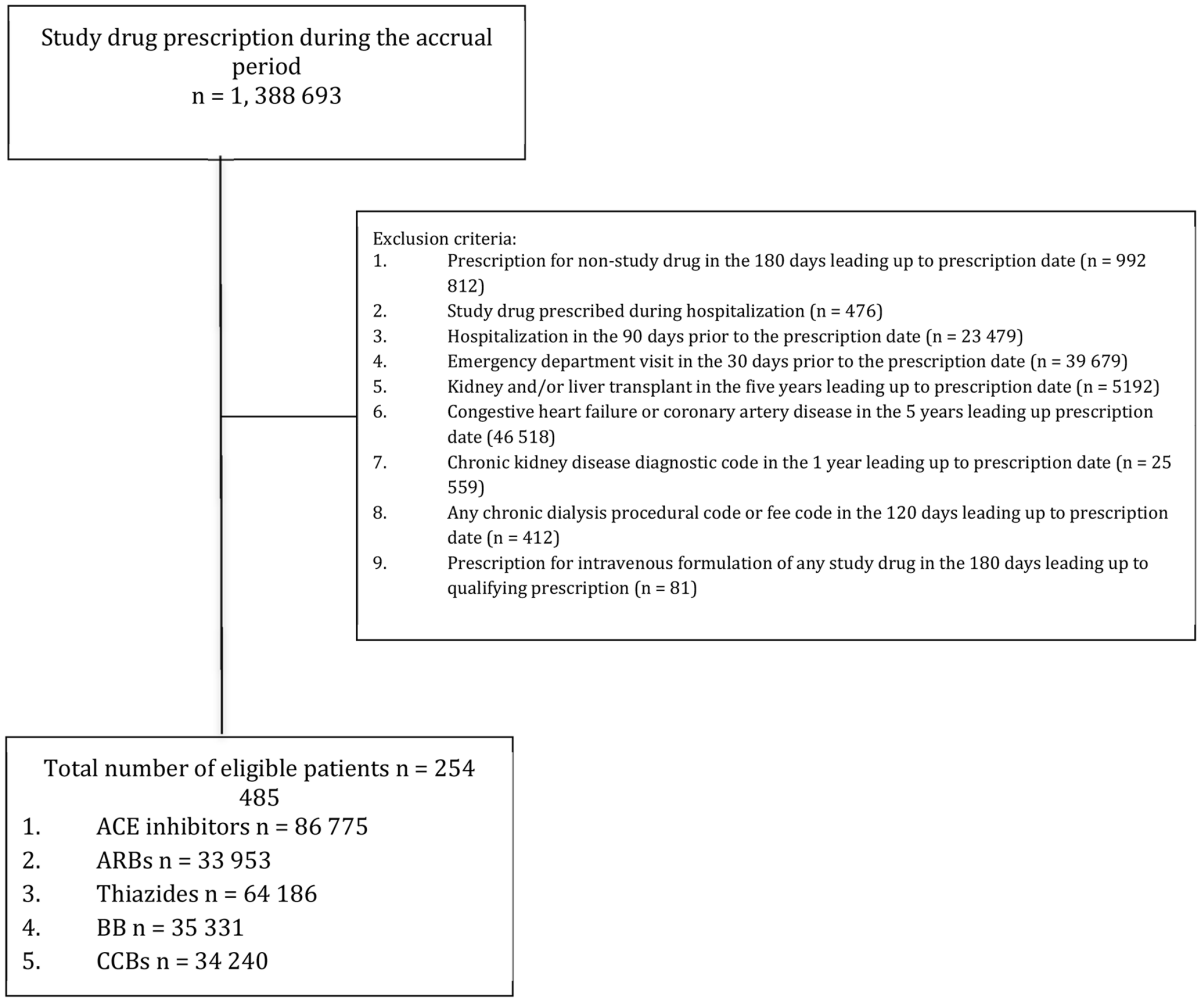
Flow diagram for the construction of our cohort.

Figure 1 shows the steps of cohort assembly. Over the study period, we identified 254 485 patients who met enrollment criteria, of whom 86 775 (34.0%) filled a prescription for an ACE inhibitor, 33 953 (13.3%) for an ARB, 34 240 (13.5%) for a CCB, 35 331 for a BB (13.9%), and 64 186 (25.2%) for a thiazide (Table 1). Mean age was 73.4 years. Total person-years follow-up for the entire cohort was 53 916.

**Table 1:** Baseline characteristics by study drug class use.

Variable		ACE		ARB		THIAZIDE		Beta Blocker		CCB		Standardized Differences^1^ (ACE vs CCB)	Standardized Differences^1^ (ARB vs CCB)	Standardized Differences^1^ (THI vs CCB)	Standardized Differences^1^ BB vs CCB)
Total Patients	N	86775		33953		64186		35331		34240					
**Female**	**N**	44988	51.84%	19181	56.49%	43742	68.15%	21411	60.60%	20617	60.21%	0.17	0.08	0.17	0.01
**Age**	**Mean (or Median)**	73.01		72.66		73.93		73.46		73.91		0.15	0.20	0.00	0.07
	**SD (or IQR)**	5.94		5.82		6.46		6.4		6.46					
	**65–69.9**	30802	35.50%	12951	38.14%	20200	31.47%	12329	34.90%	10769	31.45%	0.09	0.14	0.00	0.07
	**70–74.9**	25914	29.86%	9974	29.38%	18009	28.06%	9648	27.31%	9703	28.34%	0.03	0.02	0.01	0.02
	**75–79.9**	16868	19.44%	6339	18.67%	13035	20.31%	6849	19.39%	6855	20.02%	0.01	0.03	0.01	0.02
	**80–84.9**	8914	10.27%	3229	9.51%	8182	12.75%	4149	11.74%	4394	12.83%	0.08	0.11	0.00	0.03
	**85–89.9**	3263	3.76%	1124	3.31%	3465	5.40%	1721	4.87%	1806	5.27%	0.07	0.10	0.01	0.02
	**≥90**	1014	1.17%	336	0.99%	1295	2.02%	635	1.80%	713	2.08%	0.07	0.09	0.00	0.02
**Visits to Primary Care Providers**	**Mean (or Median)**	9.85		9.71		9.44		10.76		9.54		0.03	0.02	0.01	0.11
	**SD (or IQR)**	9.42		8.51		9.51		11.5		9.79					
	**0**	5185	5.98%	2193	6.46%	4354	6.78%	2453	6.94%	3364	9.82%	0.14	0.12	0.11	0.10
	**1–2**	9410	10.84%	3618	10.66%	7751	12.08%	3953	11.19%	4483	13.09%	0.07	0.08	0.03	0.06
	**≥3**	72180	83.18%	28142	82.89%	52081	81.14%	28925	81.87%	26393	77.08%	0.15	0.15	0.10	0.12
**Charlson Comorbidity Index Score**	**0**	76352	87.99%	30950	91.16%	58654	91.38%	31570	89.35%	31251	91.27%	0.11	0.00	0.00	0.06
	**1**	4909	5.66%	1303	3.84%	2192	3.42%	1484	4.20%	1252	3.66%	0.10	0.01	0.01	0.03
	**2**	3892	4.49%	1265	3.73%	2512	3.91%	1597	4.52%	1259	3.68%	0.04	0.00	0.01	0.04
	**≥3**	1622	1.87%	435	1.28%	828	1.29%	680	1.92%	478	1.40%	0.04	0.01	0.01	0.04
**Comorbid conditions** 2	**Chronic Liver Disease**	2195	2.53%	1031	3.04%	1349	2.10%	1018	2.88%	939	2.74%	0.01	0.02	0.04	0.01
	**Diabetes Mellitus**	22190	25.57%	4760	14.02%	2189	3.41%	2405	6.81%	2117	6.18%	0.55	0.26	0.13	0.03
	**≥1 Pneumonia in previous 5 years**	696	0.80%	196	0.58%	526	0.82%	389	1.10%	284	0.83%	0.00	0.03	0.00	0.03
	**COPD and/or chronic lung disease and/or asthma**	14377	16.57%	6020	17.73%	11254	17.53%	5332	15.09%	6101	17.82%	0.03	0.00	0.01	0.07
	**Stroke**	495	0.57%	91	0.27%	241	0.38%	272	0.77%	131	0.38%	0.03	0.02	0.00	0.05
	**Dementia**	3910	4.51%	1178	3.47%	3260	5.08%	2085	5.90%	1534	4.48%	0.00	0.05	0.03	0.06

Abbreviations: SD, standard deviation; n, number; ACE, angiotensin converting enzyme; ARB, angiotensin receptor blocker; CCB, calcium channel blocker; BB, beta blocker.

1 The standardized difference is the difference between group means expressed as a proportion of that characteristic's pooled standard deviation. It is less sensitive to sample size than hypothesis testing. Standardized differences greater than 0.1 are considered significant.

2 Comorbidities were identified using administrative codes in the five years preceding the qualifying anti-hypertensive prescription.date, chronic lung disease, stroke, dementia.

Table 1 shows the distribution of baseline characteristics across anti-hypertensive agent groups. Of the 10 baseline characteristics analyzed, six baseline characteristics were similarly distributed across the two groups, with standardized differences less than 0.1. However, women were more likely to receive a thiazide followed by a CCB and then an ACE inhibitor. Patients receiving an ACE inhibitor or ARB were slightly younger than those receiving a thiazide or CCB. Patients on an ACE inhibitor, ARB or thiazide were more likely to have seen a family doctor three or more times in the year leading up to the prescription date. Patients receiving a BB were more likely to have been prescribed a greater number of individual drugs in the year leading up to the index. Patients with diabetes were more likely to be on an ACE inhibitor or ARB compared to a CCB, and were more likely to be on a CCB compared to a thiazide.

### Primary Outcome

Results of the primary outcome are presented in Table 2. Those prescribed a CCB were used as the referent group. A total of 128 patients (0.15%) initiated on an ACE inhibitor and 43 patients (0.13%) initiated on an ARB were hospitalized with pneumonia in the subsequent 90 days. In contrast, 135 patients (0.21%) initiated on a thiazide, 112 (0.32%) of patients initiated on a BB, and 89 patients (0.26%) initiated on a CCB achieved the primary outcome. Prescription for ACE inhibitors was associated with a lower risk of hospital admission with pneumonia (adjusted OR 0.61, 95% CI0.46 to 0.81). A similar benefit was seen with ARBs (adjusted OR 0.52, 95% CI 0.36 to 0.76). In contrast, no benefit was seen with thiazides (adjusted OR 0.87, 95% CI 0.66 to 1.14) or BB (adjusted OR 1.21, 95% CI 0.91 to 1.60).

**Table 2:** Association between antihypertensive use and hospital admissions for pneumonia.

	Number of events, n (%)	Relative Risk [95% CI]	
		Unadjusted	Adjusted*
ACE inhibitor	128 (0.15%)	0.57 [0.43, 0.74]	0.61 [0.46, 0.81]
ARB	43 (0.13%)	0.49 [0.34, 0.70]	0.52 [0.36, 0.76]
Thiazide	135 (0.21%)	0.81 [0.62, 1.06]	0.87 [0.66, 1.14]
BB	112 (0.32%)	1.22 (0.92, 1.61)	1.21 (0.91, 1.60)
CCB	89 (0.26%)	1.00	1.00

Abbreviations: CI, confidence interval; ACE, angiotensin converting enzyme; ARB, angiotensin receptor blocker; CCB, calcium channel blocker; BB, beta blocker. For each analysis, patients prescribed calcium channel blockers were the referent group.

* Adjustment was by multivariable logistic regression modeling with the following variables: age, sex, primary care provider visits, Charlson comorbidity index score, chronic liver failure, pneumonia in the 5 years leading up to prescription date, chronic lung disease, stroke, dementia, diabetes.

In our sensitivity analysis, with a more conservative definition of pneumonia, the results were similar (table 3).

**Table 3:** Association between antihypertensive use and hospital admissions for pneumonia using ‘admit diagnosis’ only (sensitivity analysis).

	Number of events, n (%)	Relative Risk [95% CI]	
		Unadjusted	Adjusted
ACE inhibitor	105 (0.12%)	0.56 [0.42, 0.75]	0.60 [0.44, 0.82]
ARB	33 (0.10%)	0.45 [0.30, 0.68]	0.50 [0.33, 0.77]
Thiazide	109 (0.17%)	0.79 [0.58, 1.06]	0.84 [0.62, 1.13]
BB	84 (0.24%)	1.1 (0.81, 1.50)	1.23 (0.82, 1.54)
CCB	74 (0.22%)	1.00	1.00

Abbreviations: CI, confidence interval; ACE, angiotensin converting enzyme; ARB, angiotensin receptor blocker; CCB, calcium channel blocker; BB, beta blocker.

For each analysis, patients prescribed calcium channel blockers were the referent group. Adjustment was by multivariable logistic regression modeling with the following variables: age, sex, primary care provider visits, Charlson comorbidity index score, chronic liver failure, pneumonia in the 5 years leading up to prescription date, chronic lung disease, stroke, dementia, diabetes.

Table 4 shows the contamination rates of each study group. Contamination with another study drug occurred 15–20% of the time. Of note, Thiazides, BBs, and CCBs were most commonly contaminated by a drug from either the ACE inhibitor or ARB drug class.

**Table 4:** Rates of contamination with non-index study drugs.

Index Study Drug	ACE		ARB		THI		BB		CCB	
	n	%	n	%	n	%	n	%	n	%
Contamination with any non-index study drug during follow-up	14720	17%	5000	15%	13448	21%	5319	15%	6904	20%
Contamination with any ACE inhibitor during follow-up	N/A	N/A	560	2%	8100	13%	2214	6%	2996	9%
Contamination with any ARB during follow-up	4308	5%	N/A	N/A	1901	3%	894	3%	1381	4%
Contamination with any thiazide during follow-up	6183	7%	2313	7%	N/A	N/A	1647	5%	2645	8%
Contamination with any BB during follow-up	2193	3%	858	3%	1875	3%	N/A	N/A	1409	4%
Contamination with any CCB during follow-up	4187	5%	2165	6%	3620	6%	1717	5%	N/A	N/A

Abbreviations: CI, confidence interval; ACE, angiotensin converting enzyme; ARB, angiotensin receptor blocker; CCB, calcium channel blocker; BB, beta blocker.

### Sub-group Analysis

There was no evidence that the presence or absence of diabetes modified the association between anti-hypertensive type and 90-day hospitalization with pneumonia (p-value for interaction >.08 for each medication class). In patients without diabetes mellitus, a benefit on pneumonia rates was seen with both ACE inhibitors (adjusted OR 0.67, 95% CI 0.50 to.89) and ARBs (adjusted OR 0.49, 95% CI 0.32 to 0.75). In patients with diabetes (14.3% of the cohort), the benefit was consistent for those patients on ACE inhibitors (adjusted OR 0.29, 95% CI 0.13 to 0.65). No significant benefit was seen with ARBs (adjusted OR 0.61, 95% CI 0.27 to 1.74), recognizing that the number of events was small (ARB group n = 11 and CCB (referent) group n = 8).

### Tracer Outcome

Using CCB as the referent group, the risk of hospital admission for cholecystitis was not significantly different for patients on ACE inhibitors (adjusted OR 0.94, 95% CI 0.75 to 1.17), ARBs (adjusted OR 0.91, 95% CI 0.69 to 1.19), BBs (adjusted OR 1.10, 95% CI 0.84 to 1.40), or thiazides (adjusted OR 0.80, 95% CI 0.63 to 1.02).

## Discussion

In this population-based cohort study, we observed adult patients who were initiated on an ACE inhibitor, ARB, BB or thiazide in the outpatient setting and compared them to patients who were started on CCBs. We followed these patients for hospitalization with pneumonia within 90 days of filling their prescription. Similar to previous studies, we observed a significant reduction in pneumonia rates in patients prescribed an ACE inhibitor. In contrast to previous studies, we found that this protective effect also extended to ARBs. No such benefit was seen when thiazides and BBs were compared to CCBs.

Our study adds to the growing body of literature surrounding ACE inhibitor use and its potential to curb the incidence of pneumonia. The magnitude of benefit for ACE inhibitors was consistent with previously published reports, and in particular, a recent meta-analysis (adjusted OR 0.61 in our study vs. adjusted OR 0.66 in the meta-analysis) [6]. Interestingly, in our study both ACE inhibitors and ARBs had a fairly robust benefit with a similar adjusted OR (0.52 with ARBs). The latter is quite intriguing given the meta-analysis described above, which failed to show a reduction in the incidence of pneumonia with ARBs. As already noted, there was a discrepancy between the randomized and the two observational studies included in the meta-analysis [20], [21], and when only randomized studies were analyzed, there was a trend towards benefit with ARBs.

There are multiple potential reasons for the discrepancy between our results and the previous observational studies assessing ARB use. Etminan et al. [19] examined patients with coronary artery disease exclusively. Further, only about three percent of the cases and controls studied were taking ACE inhibitors or ARBs. Mukamal et al. [20] analyzed patients based on the anti-hypertensive drug class for which they held the longest prescription. However, patients could be on several study drug classes. As such, the benefit of ACE inhibitor and/or ARB use may have been masked as patients taking these drugs may have also been included in the control group. Lastly, both studies failed to demonstrate a protective role for ACE Inhibitors on pneumonia related hospitalization rates. As the protective effect of ACE inhibitors have been demonstrated in multiple populations, results of these two observational studies should be assessed with caution. Likely, there are issues with study design or residual confounding that have affected these studies.

There are several reasons why we are confident in our study's findings. First, the lack of benefit with thiazides and BBs provides evidence that the benefit seen is related solely to RAS blocking agents. As already mentioned, the magnitude of benefit with both ACE inhibitors and ARBs were quite similar, and in the case of ACE inhibitors, was consistent with a recent meta-analysis. It is worth mentioning that the rates of contamination were relatively small. However, given that thiazides, CCBs and BBs were most commonly contaminated RAS blocking agents, we would expect these intervening events to bias towards the null hypothesis, further bolstering our findings. Finally, our tracer outcome of cholecystitis showed no significant signal, also suggesting that residual confounding was not exerting a strong influence on our results.

### Limitations

Our study has some important limitations. As with all observational studies, the exposures we studied were not randomly assigned. While the groups were well balanced on most covariates the risk of residual confounding cannot be ruled out.

Both ACE inhibitors and ARBs reduced the incidence of pneumonia related hospitalization. The relative risk reduction for both agents was impressive, however, the absolute risk reduction and event rate were quite small. While notable, we do not feel that this negates the findings of our study given the short duration of follow-up of our study.

The population studied here was highly selected and several conditions considered to be risk factors for pneumonia were excluded. While cohort restriction is a powerful method for controlling for confounding [21], and this helped reduce indication bias, it may have implications on the generalizability of our results. However, the main comorbidities excluded in our study (i.e. patients with congestive heart failure, coronary artery disease and chronic kidney disease) leave little flexibility in the choice of anti-hypertensive medication prescribed by physicians, and RAS blockade is typically indicated. For example, in patients with congestive heart failure, existing evidence would dictate the use of RAS blocking medications to reduce morbidity and mortality. The impact of RAS blocking agents on the incidence of pneumonia is unlikely to significantly bias a prescriber's choice in such situations, given their known benefits on cardiovascular health in the heart failure population [22]. In contrast, in our selected population, where clinicians are faced with clinical equipoise, our findings may support use of RAS blocking agents over other classes of anti-hypertensives. It should be noted that while several comorbidities were excluded, a large portion of our cohort were diabetic. As diabetes is a risk factor for the development of pneumonia, and diabetic patients may preferentially be prescribed RAS blocking agents for blood pressure management, we felt that the inclusion of this group would bias towards the null hypothesis if at all. Interestingly, diabetic status did not impact the results of the primary outcome in our sub-group analysis.

Finally, there is a paucity of literature surrounding the biologic rationale for a reduction in the incidence of pneumonia with both ACE inhibitors and ARBs. While benefit from ACE inhibitors has been thought to be due to increased substance p and bradykinin levels, these levels are unaffected by ARBs [15]. As such, there must be a second, mutually exclusive rationale for the benefit observed with ARBs, or, an alternative explanation for both ACE inhibitors and ARBs. One plausible explanation is a reduction in the inflammatory response seen with both ACE inhibitors and ARBs [23]–[25]. Specifically, by reducing the inflammatory response, ACE inhibitors and ARBs may reduce the severity of pneumonia, thereby preventing pneumonia related hospitalization. Our study did not capture the incidence of pneumonia as a whole, only those severe enough to require hospitalization. Some would argue that this is the most important outcome given its association with morbidity and mortality [3].

## Conclusions

Initiation of a medication which blocks the renin angiotensin system, compared to other anti-hypertensive medications, is associated with a small 90-day reduction in the absolute risk of hospitalization with pneumonia. The magnitude of benefit seems to be similar between both ACE inhibitors and ARBs. When faced with clinical equipoise, such as in a population similar to the one studied here, physicians may preferentially prescribe one of these medications over other anti-hypertensives to help combat the morbidity associated with pneumonia. Further studies to corroborate our findings and identify potential mechanisms for this benefit are required.

## References

[1] Marrie TJ (2000) Community-acquired pneumonia in the elderly. Clin Infect Dis 31: 1066–1078 Available: http://cid.oxfordjournals.org/content/31/4/1066.full. Accessed 2013 Jun 18 11049791 10.1086/318124

[2] Almirall J, Bolíbar I, Balanzó X, González CA (1999) Risk factors for community-acquired pneumonia in adults: a population-based case-control study. Eur Respir J 13: 349–355 Available: http://www.ncbi.nlm.nih.gov/pubmed/10065680 10065680 10.1183/09031936.99.13234999

[3] Asadi L, Sligl WI, Eurich DT, Colmers IN, Tjosvold L, et al (2012) Macrolide-based regimens and mortality in hospitalized patients with community-acquired pneumonia: a systematic review and meta-analysis. Clin Infect Dis 55: 371–380 Available: http://cid.oxfordjournals.org/content/55/3/371.full. Accessed 2013 May 27 22511553 10.1093/cid/cis414

[4] Ohkubo T, Chapman N, Neal B, Woodward M, Omae T, et al (2004) Effects of an angiotensin-converting enzyme inhibitor-based regimen on pneumonia risk. Am J Respir Crit Care Med 169: 1041–1045 Available: http://www.ncbi.nlm.nih.gov/pubmed/14990394. Accessed 2013 Jun 18 14990394 10.1164/rccm.200309-1219OC

[5] Sekizawa K, Matsui T, Nakagawa T, Nakayama K, Sasaki H (1998) ACE inhibitors and pneumonia. Lancet 352: 1069 Available: http://www.thelancet.com/journals/lancet/article/PIIS0140-6736(05)60114-6/fulltext. Accessed 2013 Jun 18 10.1016/S0140-6736(05)60114-6 9759784

[6] Caldeira D, Alarcão J, Vaz-Carneiro A, Costa J (2012) Risk of pneumonia associated with use of angiotensin converting enzyme inhibitors and angiotensin receptor blockers: systematic review and meta-analysis. BMJ 345: e4260 Available: http://www.pubmedcentral.nih.gov/articlerender.fcgi?artid=3394697&tool=pmcentrez&rendertype=abstract. Accessed 2013 Jun 18 22786934 10.1136/bmj.e4260 PMC3394697

[7] Okaishi K (1999) Reduction of risk of pneumonia associated with use of angiotensin I converting enzyme inhibitors in elderly inpatients. Am J Hypertens 12: 778–783 Available: http://ajh.oxfordjournals.org/content/12/8/778.full. Accessed 2013 Jun 18 10480470 10.1016/s0895-7061(99)00035-7

[8] Vinogradova Y, Hippisley-Cox J, Coupland C (2009) Identification of new risk factors for pneumonia: population-based case-control study. Br J Gen Pract 59: e329–38 Available: http://www.pubmedcentral.nih.gov/articlerender.fcgi?artid=2751937&tool=pmcentrez&rendertype=abstract. Accessed 2013 Jun 18 19843413 10.3399/bjgp09X472629 PMC2751937

[9] Marik PE (2003) Aspiration Pneumonia and Dysphagia in the Elderly. Chest 124: 328–336 Available: http://journal.publications.chestnet.org/article.aspx?articleid=1081684&issueno=1&ijkey=c0cffa346c3a07901d8ab29aaad13ef71c3bc905&keytype2=tf_ipsecsha. Accessed 2013 May 23 12853541 10.1378/chest.124.1.328

[10] Israili ZH (1992) Cough and Angioneurotic Edema Associated with Angiotensin-Converting Enzyme Inhibitor Therapy: A Review of the Literature and Pathophysiology. Ann Intern Med 117: 234 Available: http://annals.org/article.aspx?articleid=705706. Accessed 2013 Jun 18 1616218 10.7326/0003-4819-117-3-234

[11] Tomaki M, Ichinose M, Miura M, Hirayama Y, Kageyama N, et al (1996) Angiotensin converting enzyme (ACE) inhibitor-induced cough and substance P. Thorax 51: 199–201 Available: http://thorax.bmj.com/content/51/2/199.abstract. Accessed 18 June 2013 8711657 10.1136/thx.51.2.199 PMC473042

[12] Nakayama K, Sekizawa K, Sasaki H (1998) ACE inhibitor and swallowing reflex. Chest 113: 1425 Available: http://www.ncbi.nlm.nih.gov/pubmed/9596334. Accessed 2013 Jun 18 10.1378/chest.113.5.1425 9596334

[13] Morice AH, Lowry R, Brown MJ, Higenbottam T (1987) Angiotensin-converting enzyme and the cough reflex. Lancet 2: 1116–1118 Available: http://www.ncbi.nlm.nih.gov/pubmed/2890021. Accessed 2013 Jun 18 2890021 10.1016/s0140-6736(87)91547-9

[14] Fox AJ, Lalloo UG, Belvisi MG, Bernareggi M, Chung KF, et al (1996) Bradykinin-evoked sensitization of airway sensory nerves: a mechanism for ACE-inhibitor cough. Nat Med 2: 814–817 Available: http://www.ncbi.nlm.nih.gov/pubmed/8673930. Accessed 2013 Jun 18 8673930 10.1038/nm0796-814

[15] Arai T, Yasuda Y, Takaya T, Toshima S, Kashiki Y, et al (2001) Angiotensin-converting enzyme inhibitors, angiotensin-II receptor antagonists, and pneumonia in elderly hypertensive patients with stroke. Chest 119: 660–661 Available: http://www.ncbi.nlm.nih.gov/pubmed/11171758. Accessed 2013 Jun 19 11171758 10.1378/chest.119.2.660

[16] Levy AR, O'Brien BJ, Sellors C, Grootendorst P, Willison D (2003) Coding accuracy of administrative drug claims in the Ontario Drug Benefit database. Can J Clin Pharmacol 10: 67–71 Available: http://www.ncbi.nlm.nih.gov/pubmed/12879144. Accessed 2013 Jun 19 12879144

[17] Austin PC (2009) Using the Standardized Difference to Compare the Prevalence of a Binary Variable Between Two Groups in Observational Research. Commun Stat - Simul Comput 38: 1228–1234 Available: http://dx.doi.org/10.1080/03610910902859574. Accessed 2013 Jun 19

[18] Mamdani M, Sykora K, Li P, Normand S-LT, Streiner DL, et al (2005) Reader's guide to critical appraisal of cohort studies: 2. Assessing potential for confounding. BMJ 330: 960–962 Available: http://www.bmj.com/content/330/7497/960. Accessed 2013 Jun 7 15845982 10.1136/bmj.330.7497.960 PMC556348

[19] Etminan M, Zhang B, Fitzgerald M, Brophy JM (2006) Do angiotensin-converting enzyme inhibitors or angiotensin II receptor blockers decrease the risk of hospitalization secondary to community-acquired pneumonia? A nested case-control study. Pharmacotherapy 26: 479–482 Available: http://www.ncbi.nlm.nih.gov/pubmed/16553505. Accessed 2013 Jun 19 16553505 10.1592/phco.26.4.479

[20] Mukamal KJ, Ghimire S, Pandey R, O'Meara ES, Gautam S (2010) Antihypertensive medications and risk of community-acquired pneumonia. J Hypertens 28: 401–405 Available: http://www.ncbi.nlm.nih.gov/pubmed/20051911. Accessed 2013 Jun 19 20051911 10.1097/HJH.0b013e3283330948

[21] Joffe MM, Colditz GA (1998) Restriction as a method for reducing bias in the estimation of direct effects. Stat Med 17: 2233–2249 Available: http://www.ncbi.nlm.nih.gov/pubmed/9802181. Accessed 2013 Jun 10 9802181 10.1002/(sici)1097-0258(19981015)17:19<2233::aid-sim922>3.0.co;2-0

[22] Yusuf S, Sleight P, Pogue J, Bosch J, Davies R, et al (2000) Effects of an angiotensin-converting-enzyme inhibitor, ramipril, on cardiovascular events in high-risk patients. The Heart Outcomes Prevention Evaluation Study Investigators. N Engl J Med 342: 145–153 Available: http://www.ncbi.nlm.nih.gov/pubmed/10639539. Accessed 2013 May 22 10639539 10.1056/NEJM200001203420301

[23] Hagiwara S, Iwasaka H, Matumoto S, Hidaka S, Noguchi T (2009) Effects of an angiotensin-converting enzyme inhibitor on the inflammatory response in in vivo and in vitro models. Crit Care Med 37: 626–633 Available: http://www.ncbi.nlm.nih.gov/pubmed/19114890. Accessed 2013 Jun 19 19114890 10.1097/CCM.0b013e3181958d91

[24] Gonzalez NC, Allen J, Schmidt EJ, Casillan AJ, Orth T, et al (2007) Role of the renin-angiotensin system in the systemic microvascular inflammation of alveolar hypoxia. Am J Physiol Heart Circ Physiol 292: H2285–94 Available: http://www.ncbi.nlm.nih.gov/pubmed/17208999. Accessed 2013 Jun 19 17208999 10.1152/ajpheart.00981.2006

[25] Nahmod KA, Nahmod VE, Szvalb AD (2013) Potential Mechanisms of AT1 Receptor Blockers on Reducing Pneumonia-Related Mortality. Clin Infect Dis 56: 1193–1194 Available: http://cid.oxfordjournals.org/content/56/8/1193.full. Accessed 2013 Jun 19 23315319 10.1093/cid/cit007

